# The Editor’s Choice for Issue 1, Volume 7

**DOI:** 10.3390/ijns7020031

**Published:** 2021-06-18

**Authors:** David S. Millington

**Affiliations:** Department of Pediatrics, Division of Medical Genetics, Duke University Medical Center, Durham, NC 27709, USA; dmilli@duke.edu

Dear Readers: welcome to the second issue of the Editor’s Choice, continuing the tradition started by Peter Schielen’s appraisal of Issue 4 of Volume 6 of IJNS, published in this issue [1]. Brief editorials recognizing the successful growth of our journal since its inception [2] and the contribution of reviewers during 2020 [3] are included. Apart from these editorials, the current issue consists of 17 papers, many of which belong to Special Issues. The reviews by Green [4] and Levy [5], assigned to the Special Issue on the History, Present and Future of Newborn Screening [6], are excellent and particularly relevant as we approach the birthday of Robert Guthrie, whom we regard as the “father” of newborn screening, on June 28th. Reading about Dr. Guthrie’s mass screening test for phenylketonuria (PKU) and Ann Green’s recollection of the serendipitous presence of three of the giants of newborn screening (NBS) in Birmingham, England in the early 1950s that facilitated the first dietary treatment for PKU are poignant reminders that the courage and dedication of a few committed individuals can have a profound impact on the course of human health.

Commentaries by Howell [7] and Levy [8], assigned to the Special Issue devoted to Ethical and Psychosocial Issues of NBS [9], speak to the considerable challenges raised by the prospect of expanded genomic testing in NBS. Both of these well-respected experts, who witnessed the beginnings of NBS, argue against the use of first-tier whole genome or exome sequencing, with Levy suggesting that the resulting uncertainties could threaten the standard NBS tests for inherited metabolic disorders. We look forward to further contributions to this Special Issue.

In this current issue, I was particularly impressed by two articles describing unique programs that operate outside the mainstream of NBS. First, the article by Kucera et al. [10] documents the experience of pilot screening for conditions not included in state NBS programs by means of a voluntary recruitment strategy. Known as Early Check (EC), it is a collaborative research program between Research Triangle Institute International, the North Carolina State Laboratory of Public Health (NCSLPH) and three major universities (Duke, North Carolina at Chapel Hill and Wake Forest). The objective of EC is to demonstrate the feasibility and acceptability of statewide screening and follow-up and to generate valuable information to support nominations for conditions not yet approved for NBS. Screening for spinal muscular atrophy (SMA) began in October 2018, before SMA was added to the Recommended Uniform Screening Panel (RUSP) in the United States. Fragile X syndrome and, more recently, Duchenne Muscular Dystrophy are also included in the program, which uses a variety of recruitment strategies pre- and post-partum, including mailings to mothers of all newborns who were screened by the state, invitations with a link to a recruitment portal via social media, paid advertisements and distribution of informational material through select hospitals and clinics. These strategies have met with limited degrees of success, with an overall recruitment rate of only about 5% of the newborn population in the state. However, all but one of the counties across the state are represented, mitigating potential regional bias. Over a 2-year period, SMA screening was performed on 12,065 newborns using a real-time qPCR assay to detect the presence or absence of the homozygous deletion of *SMN1* at exon 7, which is common to >95% of patients affected by SMA. Of these, two were reported as screen-positive and two gave unsatisfactory results, while the rest were normal. Of the two screen-positive cases, one was borderline and ultimately diagnosed with a rare genetic disorder that causes neutropenia, and the other was a clear positive confirmed to have SMA, who is now receiving treatment. This study highlights the challenges of managing an opt-in NBS pilot study without direct access to the mothers. Despite this limitation, EC’s unique virtual recruitment strategy has enabled a successful pilot NBS for SMA to be undertaken and has afforded valuable information to the NCSLPH (and to other NBS programs) prior to statewide screening by mandate.

The second article is a very impressive contribution from the only neonatal screening program in the world that utilizes urine (Auray-Blais et al. [11]). This voluntary screening program in Quebec, Canada has operated continuously since its inception almost 50 years ago, employing thin-layer chromatography (TLC) with spray-reagents that can detect up to 25 inherited metabolic diseases (IMDs). Since 1981, dried urine specimens (DUS) on filter paper were collected at home by the parents from newborns at 21 days of age and mailed to the laboratory for analysis. The urine screening program began and has run concurrently with the dried blood spot (DBS) NBS program, with the objective of identifying conditions not reliably detectable in DBS at 1–2 days of age prior to the onset of symptoms, some of which have a high prevalence in French–Canadians. The compliance rate of ~90% is truly remarkable for a voluntary NBS program. In their paper, the authors describe new methods based on liquid chromatography–tandem mass spectrometry (LC-MS/MS) to replace TLC. The initial screening test is performed on DUS extracts by flow-injection MS/MS using stable isotope-labeled internal standards for quantitative analysis of 22 biomarkers, normalized to creatinine, that target methylmalonic and several other organic acidurias, urea cycle disorders including hyperornithinemia–hyperammonemia–homocitrullinuria (HHH) syndrome, as well as cystinuria, homocystinuria and disorders of creatine synthesis and transport, with a cycle time of about 2.8 min. Presumptive positives are then reflexed to second-tier testing by ultra-performance liquid chromatography (UPLC)-MS/MS assays, also using stable isotope labeled internal standards that target amino acids and organic acids in the same DUS extracts used for the initial screen.

This manuscript should be of particular interest to biochemical genetics laboratories engaged in the follow-up of abnormal NBS screens that currently use amino acid analyzers and gas chromatography/mass spectrometry (GC/MS) for analysis of amino acids and organic acids in urine, respectively. The authors developed separate, fully validated UPLC-MS/MS methods for their targeted analysis of organic and amino acids and compared them rigorously with existing methods based on amino acid analyzers and GC/MS. The cycle times for these robust new assays are both approximately 8 min, which is much shorter than the alternative methods. Furthermore, because biochemical genetics laboratories currently use LC-MS/MS for analysis of acylcarnitines and other biomarkers, they could consider amalgamating all of these valuable diagnostic tests onto a single platform.
